# Sensation-Seeking and Impulsivity as Predictors of Reactive and Proactive Aggression in Adolescents

**DOI:** 10.3389/fpsyg.2016.01447

**Published:** 2016-09-27

**Authors:** María Del Carmen Pérez Fuentes, Maria del Mar Molero Jurado, José J. Carrión Martínez, Isabel Mercader Rubio, José J. Gázquez

**Affiliations:** University of Almería, AlmeríaSpain

**Keywords:** sensation-seeking, impulsivity, proactive aggression, reactive aggression, adolescent

## Abstract

In adolescence, such matters as substance use and impulsiveness may give rise to problematic behavior repertoires. This study was therefore done to analyze the predictive value of sensation-seeking and impulsiveness dimensions related to the functions of aggression (reactive/proactive) and types of expression (physical/relational). A total of 822 high school students in Almeria (Spain) aged 13–18, were administered the Sensation-Seeking Scale, the State Impulsiveness Scale and Peer Conflict Scale. The results show the existence of a positive correlation of the majority of factors analyzed, both in impulsiveness and sensation-seeking, with respect to the different types of aggression. Furthermore, aggressive behavior is explained by the combination of a sensation-seeking factor (Disinhibition) and two impulsiveness factors (Gratification and Automatism). This study shows the need to analyze aggression as a multidimensional construct.

## Introduction

Adolescence is a stage of change in which the individual must make decisions and respond to a diversity of situations. Such matters as substance use (Gázquez et al., 2015a) or how to interact with others (Inglés et al., 2014) could thus become repertoires of problem behavior in adolescents. In addition to individual factors (Gázquez et al., 2015b; Valle et al., 2015), adopting certain risk and/or problem behaviors also depends on other family (Martínez-Loredo et al., 2016) or peer group (Monahan et al., 2009) factors which are determining for the construction of self-concept and personal wellbeing (Nacimiento and Mora-Merchán, 2014; Álvarez et al., 2015; Azpiazua et al., 2015; Goñi et al., 2015).

Adolescence is also characterized by premature experimentation of new experiences and sensations. Jessor et al. (1998) argue that sensation-seeking can interfere with healthy adolescent development, and has been shown to be one of the most important risk factors in behavior problems. For many adolescents, the social setting inhibits imprudence, but for others it promotes risk-taking and emotion-seeking. These experiences sometimes include drug use, with negative consequences for their development that later become evident (Pérez-Fuentes et al., 2015). MacPherson et al. (2010) found that assuming risks was an important predictor of adolescent drinking. Curran et al. (2010) observed that adolescents who drive while under the effects of alcohol were strongly correlated with sensation-seeking factors, specifically with emotions and excitement, disinhibition and susceptibility to boredom.

An important matter related to the increase in sensation-seeking during adolescence and also aggressive behavior is impulsivity (Archer and Webb, 2006). This is defined as an expression of uninhibited behavior characterized by lack of control of behavior (Cardoso-Moreno et al., 2015). Given the role of impulsivity in involvement in risk behaviors by adolescents, the positive effect of interventions during childhood to prevent the first forms of impulsivity, which continue into adolescence if not treated, is clear.

Impulsivity and aggression maintain a pattern of consistent relationship. However, not everyone who is impulsive has aggressive behavior, nor is it manifested in the same way. For example, Hatfield and Dula (2014) found that high scores on impulsivity were associated with higher levels of physical or direct aggression. Grimaldi et al. (2014) suggest that relational aggressors may be more exposed to negative consequences related to alcohol when they respond impulsively to negative emotions.

Aggression, currently conceived as a multidimensional construct, can take many forms (García-Sancho et al., 2016). The functions of aggression refer to the aggressor’s motivation, and historically, two types have been distinguished, proactive and reactive (Hartup, 1974; Dodge and Coie, 1987). Proactive aggression refers to deliberate actions directed at achieving a goal, while reactive aggression refers to emotional response to attack. Thus different theoretical approaches postulate the intervention of different cognitive and social processes for each function (Gifford-Smith and Rabiner, 2004). The problem in detecting the relationships of the different aspects of proactive and reactive aggression is that the measures of these two functions are often intercorrelated (Dodge et al., 1990). However, later studies have shown that the two functions of aggression behave like two independent constructs (Poulin and Boivin, 2000), although they often occur at the same time (Bushman and Anderson, 2001). For example, reactive aggression is often related to problems with emotional regulation, internalization symptoms, rejection by classmates or victimization (Card and Little, 2006). In some cases this type of effect is seen in youth who reject school (Inglés et al., 2015). Subjects who show proactive aggression overestimate the positive consequences of aggression and minimize the probability of being punished for it (Marsee and Frick, 2007). In this respect, scientific evidence suggests that there is a relationship between proactive aggression and certain traits of insensitivity and lack of empathy or guilt (Frick and Dickens, 2006).

Thus both reactive and proactive aggression have been associated with negative effects for development of the individual (Hubbard et al., 2010), even with consequences in later stages (Cleverley et al., 2012). For example, such consequences as anxiety and depression in reactive aggressors (Fite et al., 2014) or the proliferation of antisocial/delinquent behavior with impulse control in proactive aggressors (Scarpa et al., 2010) have been observed.

Sensation-seeking has been related to the development of aggressive behavior (Wilson and Scarpa, 2011). Risk-taking, as proneness to acting impulsively to achieve reward even though there are negative consequences, would also be associated with aggression and delinquency (Romer, 2010). Previous studies on the relationship between personality and antisocial behavior have shown that both failure to control impulses and sensation-seeking are related to aggression and rule-breaking (Newcomb and McGee, 1991). Similarly, little inhibition in childhood leads to rule-breaking and becomes a risk factor for aggression in adolescents (Moeller et al., 2001).

Raine et al. (2006) found that low levels of inhibition and high sensation-seeking were present in adolescents with both reactive and proactive aggression. Findings such as these report the association between impulsive tendencies and the reactive and proactive forms of aggression. However, proactive aggressive individuals can show a stronger ability to regulate immediate aggressive impulses, channeling them into planned aggression (Dodge et al., 2006). Other authors, such as Steinberg et al. (2008) observed specific effects (not general) of sensation-seeking on deviant behavior and attitudes in adolescents who years before has used drugs and/or had participated in delinquent activities.

Recently, authors like Cui et al. (2016) have concluded that children whose high levels of reactive and proactive aggression persisted over time also had high levels of sensation-seeking and risk-taking, as well as low levels of moral reasoning.

Moreover, two forms of aggression related to interpersonal relationships may be distinguished (Buss, 1961; Valzelli, 1983; Grotpeter and Crick, 1996), physical (direct) and relational (indirect) aggression. Some studies (Stickle et al., 2012) mention that males and females show signs of relational aggression in different ways. Centifanti et al. (2015) suggested that individual factors are associated with participation in relational aggression by girls and may therefore be an indicator of problem behavior.

The purpose of this study was to find out the predictive value of variables related to sensation-seeking and impulsivity related to the functions of aggression (reactive/proactive) and its forms of expression (physical/relational) in adolescents.

## Materials and Methods

### Participants

A sample of 822 high school students was selected by cluster sampling from eight high schools in the province of Almeria (Spain). The participants were aged 13–18 with a mean of 14.84 years (*SD* = 0.87). Of the total simple, 51.8% (*n* = 426) were males and 48.2% (*n* = 396) females, with mean ages of 14.85 (*SD* = 0.87) and 14.82 years (*SD* = 0.86), respectively. The distribution of our sample by academic year was: 43.7% were 3rd year high school students (*n* = 359) and the remaining 56.3% were in 4th year (*n* = 463).

### Instruments

*Escala de Búsqueda de Sensaciones* [*Sensation-Seeking Scale*] (Pérez and Torrubia, 1986). This scale measures the tendency to seek new risky experiences. It consists of a total of 40 items with dichotomous answers (yes/no) on four subscales: Emotion-seeking (BEM), Excitement-seeking (BEX), Disinhibtion (DES) and Susceptibility to Boredom (SAB). The authors found reliability coefficients over 0.87 for the whole questionnaire scale and Cronbach’s alpha of 0.70–0.87 on the subscales.

*Escala de Impulsividad Estado* [*State Impulsivity Scale*] (EIE; Iribarren et al., 2011). This scale was developed to evaluate impulsive behavior defined as a state, that is, impulsivity as a manifest behavior that can vary in the short term. It consists of 20 items, with a response format based on a four-point Likert-type scale in which the subject is asked to evaluate the frequency with which each of the statements is true. The items that make up the scale are grouped into three subscales: Gratification (urgency in satisfying impulses, preference for immediate reward, intolerance to frustration and tendency to act without thinking of negative consequences); Automatism (repeated, rigidly expressed behavior, with no attention to contextual variables); and Attentional (presence of unplanned behavior which takes place too soon without considering all the information available). The authors found high reliability for the complete scale (α = 0.88) and each of its dimensions: Gratification (*α* = 0.84), Automatism (*α* = 0.80) and Attentional (*α* = 0.75).

*Peer Conflict Scale* (PCS; Marsee et al., 2004). This is a self-report scale developed to evaluate the forms and functions of aggression. It consists of 40 items distributed among four subscales: Physical Proactive Aggression, Physical Reactive Aggression, Relational Proactive Aggression and Relational Reactive Aggression. The response format is based on a four-point Likert-type scale (0 = not at all true, 1 = somewhat true, 2 = very true and 3 = definitely true), and the elements are grouped in four subscales, scoring 0–30 on each. The authors (Marsee et al., 2011), found satisfactory internal consistency for each of the subscales (Physical Reactive α = 0.87; Reactive Relational α = 0.77; Physical Proactive α = 0.79; Relational Proactive α = 0.76).

### Procedure

This study was exempt from ethical approval, because the study did not involve any potential risk for the participants. All participants provided written consent. Before collecting data, a meeting was held with the school directors/counselors where they were informed of the purposes, procedure and use of research data. When the tests were administered, the participants were guaranteed confidential data processing and given instructions for their completion. They were also informed that they were voluntary, anonymous and that their data were protected by applicable legislation. Two members of the research team traveled to the high schools to administer the tests.

### Data Analysis

First, to identify the variables related to the different forms of aggression analyzed (Physical Proactive, Physical Reactive, Relational Proactive, and Relational Reactive), the Pearson’s correlation coefficient was calculated as well as the corresponding descriptive statistics.

Stepwise multiple linear regression analysis was performed to find out how the predictor variables (Sensation-seeking: Gratification, Automatism and Attentional; Impulsivity: Emotion-seeking, Excitement-seeking, Disinhibition and Susceptibility to boredom) were related to each of the criterion variables.

## Results

### Sensation-Seeking and Impulsivity Factors Related to Forms of Aggression

The correlation coefficients found show the existence of positive correlations between impulsivity (Gratification, Automatism and Attentional) and sensation-seeking (Emotion-seeking, Excitement-seeking, Disinhibition and Susceptibility to boredom) factors and the forms of aggression analyzed.

As observed in **Table 1**, adolescents with high scores in physical proactive aggression (AgPA) showed high levels of Gratification (*r* = 0.31; *p* < 0.001), Automatism (*r* = 0.26; *p* < 0.001), Attentional impulsivity (*r* = 0.24; *p* < 0.001), Emotion-seeking (*r* = 0.06; *p* < 0.05), Disinhibition (*r* = 0.35; *p* < 0.001) and Susceptibility to boredom (*r* = 0.17; *p* < 0.001).

**Table 1:** Descriptive statistics of impulsivity and sensation-seeking factors and correlation coefficients with form of aggression (*N* = 822).

	Mean	*SD*	Correlation with AgPA	Correlation with AgPR	Correlation with AgRA	Correlation with AgRR
**Impulsivity**						
GRA	13.19	4.00	0.31***	0.30***	0.35***	0.31***
AUTO	11.86	4.08	0.26***	0.26***	0.32***	0.26***
ATEN	14.14	4.35	0.24***	0.24***	0.32***	0.25***
**Sensation-seeking**						
BEM	6.10	2.57	0.06*	0.04	0.13***	0.07*
BEX	5.24	1.72	0.04	0.02	0.09**	0.05*
DES	4.34	2.30	0.35***	0.31***	0.40***	0.31***
SAB	4.22	1.95	0.17***	0.16***	0.22***	0.19***

*Significant correlation at 0.05; **Significant correlation at 0.01; ***Significant correlation at 0.001. AgPA, Physical Proactive Aggression; AgPR, Relational Proactive Aggression; AgRA, Physical Reactive Aggression; AgRR, Relational Reactive Aggression; GRA, Gratification; AUTO, Automatism; ATEN, Attentional; BEM, Emotion-seeking; BEX, Excitement-seeking; DES, Disinhibition; SAB, Susceptibility to boredom.

Furthermore, high scores on Relational Proactive Aggression (AgPR) correlated positively with all the Impulsivity factors (Gratification: *r* = 0.30; *p* < 0.001; Automatism: *r* = 0.26; *p* < 0.001; Attentional: *r* = 0.24; *p* < 0.001), Disinhibition (*r* = 0.31; *p* < 0.001) and Susceptibility to boredom (*r* = 0.16; *p* < 0.001). Physical Reactive Aggression (AgRA) as a component of aggression may be seen in **Table 1** to be correlated with all the components of Impulsivity (Gratification: *r* = 0.35; *p* < 0.001; Automatism: *r* = 0.32; *p* < 0.001; Attentional: *r* = 0.32; *p* < 0.001 and sensation-seeking (Emotion-seeking: *r* = 0.13; *p* < 0.001; Excitement-seeking: *r* = 0.09; *p* < 0.01; Desinhibition: *r* = 0.40; *p* < 0.001; Susceptibility to boredom: *r* = 0.22; *p* < 0.001).

Finally, as shown by the correlation coefficients found for Relational Reactive Aggression (AgRR), adolescents who showed this form of aggression were also observed to have high scores on Gratification (*r* = 0.31; *p* < 0.001), Automatism (*r* = 0.26; *p* < 0.001), Attentional (*r* = 0.25; *p* < 0.001), Emotion-seeking (*r* = 0.07; *p* < 0.05), Excitement-seeking (*r* = 0.05; *p* < 0.05), Desinhibition (*r* = 0.31; *p* < 0.001) and Susceptibility to boredom (*r* = 0.19; *p* < 0.001).

### Proactive Aggression (Physical/Relational) Predictor Variables

Regression analysis yielded three models for Physical Proactive Aggression (**Table 2**) of which Model 3 has the most explanatory power with 16.2% (*R^2^* = 0.16) of the variance explained by the factors included in the model.

**Table 2:** Stepwise multiple linear regression (Physical Proactive Aggression).

Model	*R*	*R^2^*	Corrected *R^2^*	Change statistics				Durbin Watson
				Standard error of estimate	Change in *R^2^*	Change in *F*	Significance of change in *F*	
1 (DES)	0.35	0.12	0.12	3.75	0.12	116.41	0.00	1.78
2 (DES, GRA)	0.39	0.15	0.15	3.69	0.02	28.05	0.00	
3 (DES, GRA, AUTO)	0.40	0.16	0.15	3.67	0.00	8.45	0.00	

**Model**		**Unstandardized coefficients**		**Standardized coefficients**	***T***	**Sig.**	**Collinearity**	
		***B***	**Standard error**	**Beta**			**Tol.**	**VIF**

(Constant)		-2.47	0.47		-5.26	0.00		
DES		0.44	0.06	0.25	7.01	0.00	0.76	1.30
GRA		0.12	0.04	0.12	2.99	0.00	0.55	1.80
AUTO		0.11	0.04	0.11	2.90	0.00	0.65	1.51

*DES, Disinhibition; GRA, Gratification; AUTO, Automatism.*

To confirm model validity, independence of residuals was analyzed. The Durbin–Watson *D* statistic found was *D* = 1.78, which confirms the absence of positive and negative autocorrelation.

As shown in **Table 2**, the *T* was associated with a probability of error below 0.05 in all the variables included in the model. Furthermore, the standardized coefficients revealed that the variables with the most explanatory weight were Disinhibition, Automatism and Gratification, and the first of them (Disinhibition) was the strongest predictor of Physical Proactive Aggression. Finally, the absence of collinearity between variables included in the model may be assumed as tolerance is high and VIF is low.

The three Relational Proactive Aggression models resulting from the regression analysis are shown in **Table 3**, where Model 3 found 14.1% explained variance (*R*^2^ = 0.14). In this case, the Dubin–Watson *D* confirmed no correlation of residuals (*D* = 1.68).

**Table 3:** Stepwise multiple linear regression (Relational Proactive Aggression).

Model	*R*	*R^2^*	Corrected *R^2^*	Change statistics				Durbin Watson
				Standard error of estimate	Change in *R^2^*	Change in *F*	Significance of change in *F*	
1 (DES)	0.31	0.10	0.10	3.54	0.10	92.21	0.00	1.68
2 (DES, AUTO)	0.36	0.13	0.13	3.47	0.03	30.73	0.00	
3 (DES, AUTO, GRA)	0.37	0.14	0.13	3.46	0.00	7.08	0.00	

**Model**		**Unstandardized coefficients**		**Standardized coefficients**	***T***	**Sig.**	**Collinearity**	
		***B***	**Standard error**	**Beta**			**Tol.**	**VIF**

(Constant)		-1.88	0.44		-4.25	0.00		
DES		0.36	0.06	0.22	6.01	0.00	0.76	1.30
AUTO		0.12	0.03	0.13	3.33	0.00	0.65	1.51
GRA		0.10	0.04	0.11	2.66	0.00	0.55	1.80

*DES, Disinhibition; GRA, Gratification; AUTO, Automatism.*

The *T* statistic shows an association with a probability of error below 0.05 for all the variables included in the model, Gratification, Desinhibition and Automatism. According to the standardized coefficients found in this case, the Disinhibition variable is the strongest predictor of relational proactive aggression.

In view of the values found for the Tolerance and VIF indicators, in this case, collinearity of variables is assumed to be absent, since tolerance is high and VIF is low.

### Reactive Aggression (Physical/Relational) Predictor Variables

As shown in **Table 4**, three models were found when Physical Reactive Aggression was the variable entered, the third of which had the most explanatory power. The Automatism, Gratification and Disinhibition variables included in the model explained 21.5% of the variance (*R^2^* = 0.21) in physical reactive aggression. The validity of the model is also reflected in the independence of the residuals with a Durbin–Watson *D* = 1.85.

**Table 4:** Stepwise multiple linear regression (Physical Reactive Aggression).

Model	*R*	*R^2^*	Corrected *R^2^*	Change statistics				Durbin Watson
				Standard error of estimate	Change in *R^2^*	Change in *F*	Significance of change in *F*	
1 (DES)	0.40	0.16	0.16	5.03	0.16	159.96	0.00	1.85
2 (DES, AUTO)	0.45	0.21	0.20	4.89	0.04	48.89	0.00	
3 (DES, AUTO, GRA)	0.46	0.21	0.21	4.87	0.00	7.76	0.00	

**Model**		**Unstandardized coefficients**		**Standardized coefficients**	***T***	**Sig.**	**Collinearity**	
		***B***	**Standard error**	**Beta**			**Tol.**	**VIF**

(Constant)		-2.46	0.62		-3.95	0.00		
DES		0.71	0.08	0.29	8.41	0.00	0.76	1.30
AUTO		0.24	0.05	0.17	4.50	0.00	0.65	1.51
GRA		0.15	0.05	0.11	2.78	0.00	0.55	1.80

*DES, Disinhibition; GRA, Gratification; AUTO, Automatism.*

The *T* statistic is associated with a probability of error of less than 0.05 in the five variables in the model (**Table 4**). And according to the standardized coefficients, the variables with the highest explanatory weight are Disinhibition, Automatism and Gratification. These coefficients show the Disinhibition variable to be the strongest predictor of physical reactive aggression. In this case, collinearity of variables is assumed to be absent as indicated by high tolerance and low VIF.

As the result of multiple regression analysis, three models are also found for Relational Reactive Aggression, of which Model 3 is the one with the highest explanatory power, with 14.4% (*R^2^* = 0.14) of the variance explained by the factors included in the model (**Table 5**).

**Table 5:** Stepwise multiple linear regression (Relacional Reactiva Agresión).

Model	*R*	*R^2^*	Corrected *R^2^*	Change statistics				Durbin Watson
				Standard error of estimate	Change in *R^2^*	Change in *F*	Significance of change in *F*	
1 (DES)	0.31	0.10	0.10	3.96	0.10	91.98	0.00	1.65
2 (DES, GRA)	0.36	0.13	0.13	3.88	0.03	32.63	0.00	
3 (DES, GRA, AUTO)	0.38	0.14	0.14	3.86	0.00	8.60	0.00	

**Model**		**Unstandardized coefficients**		**Standardized coefficients**	***T***	**Sig.**	**Collinearity**	
		***B***	**Standard error**	**Beta**			**Tol.**	***VIF***

(Constante)		-1.27	0.49		-2.57	0.01		
DES		0.38	0.06	0.21	5.76	0.00	0.76	1.30
GRA		0.14	0.04	0.14	3.33	0.00	0.55	1.80
AUTO		0.12	0.04	0.11	2.93	0.00	0.65	1.51

*DES, Disinhibition; GRA, Gratification; AUTO, Automatism.*

To confirm the validity of the model, the independence of residuals was analyzed. The Durbin–Watson *D* was *D* = 1.65, confirming absence of positive and negative autocorrelation.

**Table 5** shows that the *T* value is associated with a probability of error of less than 0.05 in all the variables included in the model. Furthermore, the standardized coefficients reveal that the Disinhibition factor is the strongest predictor of relational reactive aggression.

Finally, collinearity of the variables included in the model is assumed to be absent since tolerance indicators are high and VIF is low.

## Discussion and Conclusion

In the first place, results show the existence of a positive correlation of most of the impulsivity and sensation-seeking factors analyzed with the different forms of aggression (Newcomb and McGee, 1991; Raine et al., 2006). The exceptional cases in which there is no correlation refer to proactive aggression and emotion/excitement-seeking. It is reasonable to argue that sensation-seeking and risk-taking become more evident in children with reactive aggression, since they tend to seek excitement and act on their impulses in the rush of the moment, while children with proactive aggression are able to channel their aggressive behavior in a more calculated manner (Dodge et al., 2006). According to Steinberg et al. (2008), sensation-seeking could have specific effects on deviant behavior. Thus the expression of a certain type of aggression could be mediated by individual factors (Centifanti et al., 2015; Gázquez et al., 2015b), cognitive and social processes (Gifford-Smith and Rabiner, 2004; Monahan et al., 2009; Martínez-Loredo et al., 2016) and even characterization of the various expressions of aggression itself (Stickle et al., 2012).

The purpose of this study was to find out the predictive value of sensation seeking and impulsivity for the functions of aggression (reactive/proactive) and its forms of expression (physical/relational) in adolescents. The results of multiple regression analysis reveal that in all cases, aggressive behavior is explained by the combination of a sensation-seeking factor (Disinhibition) and two impulsivity factors (Gratification and Automatism). The fact that they are the same components that combine to construct the model of each of the forms of aggression analyzed (García-Sancho et al., 2016) supports the idea of interrelation of the functions of aggression (Dodge et al., 1990).

The presence of the Disinhibition component as one of the aggression predictor variables (Wilson and Scarpa, 2011) demonstrates the tendency to experience new sensations during adolescence (Jessor et al., 1998). This orientation toward sensation-seeking is present not only in the development of aggressive behavior, but is also found as a predictor in other adolescent problem behavior such as substance use (Curran et al., 2010). The inclusion of impulsivity factors, which are also present in other adolescent risk behaviors (MacPherson et al., 2010), in the models reflects their association with aggression in all its forms (Moeller et al., 2001; Romer, 2010).

Relatively few studies have considered sensation-seeking and impulsivity in relation to reactive and proactive aggression (Cui et al., 2016). Findings such as these show the need to give the analysis of aggression as a multidimensional construct more attention (García-Sancho et al., 2016) to the extent that its different functions and forms of expression have been identified as responsible for a variety of effects in adolescent development (Hubbard et al., 2010; Scarpa et al., 2010; Fite et al., 2014), and even afterward (Cleverley et al., 2012).

The main limitations of the study are that: (1) The sample, although representative, is comprised only of high school students and cannot be generalized to other grade levels, and therefore, one of the future lines of research is the replication of this study in other years; and, (2) the biases typical of self-report techniques, for example, the associations found with the effects of social desirability, which with age show positive relationships to certain desirable characteristics in the self-report.

However, although this study does have some limitations which should be kept in mind for future research, it may be considered a precursor, and is of great interest for the relevant data it contributes to the design of interventions which make it possible to work on reducing risk factors and strengthening those which protect against aggressive behavior.

Therefore, progress made in research along this line requires development of future analyses based on causal models and the analysis of mediating factors in aggressive behavior in all of its forms. In other words, adolescent intervention must be able to counteract the tendency to sensation-seeking and any other form of impulsivity related to the origin and/or maintenance of aggressive behavior.

## Author Contributions

MP and MJ (Drafting and analysis of data). JM and IR (bibliographic search). JG (Reviewers made changes).

## Conflict of Interest Statement

The authors declare that the research was conducted in the absence of any commercial or financial relationships that could be construed as a potential conflict of interest.
